# Fragmentation of Chitosan by Acids

**DOI:** 10.1155/2013/508540

**Published:** 2013-11-02

**Authors:** Mohammad Reza Kasaai, Joseph Arul, Gérard Charlet

**Affiliations:** ^1^Faculty of Agricultural Engineering, Sari Agricultural Sciences and Natural Resources University, Khazar Abad Road, Km. 9, P.O. Box 578, Sari, Mazandaran, Iran; ^2^Department of Food Science and Nutrition, Université Laval, Sainte-Foy, Quebec, QC, Canada G1K 7P4; ^3^Department of Chemistry, Université Laval, Sainte-Foy, Quebec, QC, Canada G1K 7P4

## Abstract

Fragmentation of chitosan in aqueous solution by hydrochloric acid was investigated. The kinetics of fragmentation, the number of chain scissions, and polydispersity of the fragments were followed by viscometry and size exclusion chromatography. The chemical structure and the degree of N-acetylation (DA) of the original chitosan and its fragments were examined by ^1^H NMR spectroscopy and elemental analysis. The kinetic data indicates that the reaction was of first order. The results of polydispersity and the DA suggest that the selected experimental conditions (temperature and concentration of acid) were appropriate to obtain the fragments having the polydispersity and the DA similar to or slightly different from those of the original one. A procedure to estimate molecular weight of fragments as well as the number of chain scissions of the fragments under the experimental conditions was also proposed.

## 1. Introduction 

**Figure 1:**
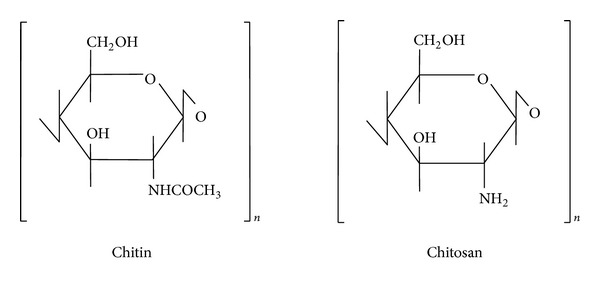
Chemical structure of chitin and chitosan.

Chitin is the second most abundant polysaccharide in nature, only after cellulose. Its chemical structure is similar to cellulose: both polysaccharides have *β*-(1, 4) glycosidic linkages and are able to form intermolecular hydrogen bonds. Chitin is highly crystalline and insoluble in common solvents. It is often converted into chitosan by deacetylation which renders it soluble in acids. The word “chitosan” is used for both partially and completely N-deacetylated chitosans. Commercial chitin and chitosan are copolymers of 2-acetamido-2-deoxy-D-glucose (N-acetyl glucosamine, GlcNAc: A-unit) and 2-amino-2-deoxy-D-glucose (glucosamine, GlcNH_2_: D-unit) with *β*-D-(1 → 4) glycoside linkages. The chemical structures of chitin and chitosan are illustrated in Figure 1.

Chitosan with vast and diverse interesting (antifungal, antibacterial, scavenging, and antioxidant activities; elicitation of plant defense; cholesterol lowering effect; wound healing; and film and fiber forming) properties has potential for applications in many areas such as food, medicine, and agriculture [1–5]. It has been employed for various applications in food industries as an antimicrobial agent; as a feed supplement, food additive, and food preservative; purification agent for water and extends shelf life of fresh fruits and vegetables [2, 4, 6–15]. 

The various properties and biological activity of chitosan and its derivatives appear to be dependent on their molecular weight; molecular weight distribution; and DA [16–19]. Low molecular weight chitosans have been used as food coating materials [4, 17, 19]. Intermediate molecular weights of chitosan (100 < *M*_*w*_ < 500 kDa) and chitosan-based polysaccharides have been used in food industry due to their film forming ability [4, 14]. The biological and antimicrobial activities and effectiveness of chitosan or its derivatives have been found to be dependent on its degree of acetylation (DA) and molecular weight in various applications [16–20]. The use of chitosan in many applications is in the form of solution at moderate concentration and using intermediate molecular weights [21]. 

Large macromolecules of chitosans are less effective or develop high solution viscosities, which can cause practical problems in their applications. Development of an efficient process for fragmentation and reduction in molecular size of chitosan without altering its DA is interestingand a desirable goal. Chitosan can be fragmented by acid or enzymatic hydrolysis. Acid hydrolysis has an advantage over enzymatic hydrolysis, because enzymes are expensive. Acid hydrolysis is a convenient way to obtain a wide range of fragments of different molecular weights by changing the reaction time and/or acid concentration. Acid hydrolysis of chitosan has been studied by several researchers. In most of the reports, studies were focused on depolymerization of oligomers and/or depolymerization of chitosan for preparation of small macromolecules such as chitooligosaccharides [22–25] using high concentration of acids (*C*_HCl_ > 1 N). These studies resulted in oligomers, monomers, or macromolecules having larger polydispersities compared to the original ones. The DA of the fragments altered during fragmentation process. Studies on depolymerization of chitosans having relatively high initial molecular weights by acid hydrolysis have been also reported [26–28]. 

The present studies focused on fragmentation of chitosan for preparation of the fragments having wide ranges of molecular weights using soft experimental conditions (concentration of HCl between 0.1 and 1 M and temperature = 65°C). We report the effect of HCl (*C* = 0–1.0 N) on the average number of chain scissions (*α*), molecular weight distribution (polydispersity), and the DA of the fragments. A rapid method to estimate molecular weight of fragments while fragmentation process is continuing is proposed. 

## 2. Materials and Methods

### 2.1. Materials

High viscosity shrimp-shell chitosan with a nominal DA of 25% was purchased from Nova-Chem. Ltd. (Halifax, Nova Scotia, Canada) and was purified as follows: chitosan was dissolved in 0.2 M HCl with constant stirring for 16 hours at room temperature. Suspended particles remaining in the solution were separated by centrifugation. The solutions were neutralized (pH, 7.8) with 1.0 M NaOH. The chitosan particles suspended in aqueous medium were recovered by centrifugation, washed with deionized water several times, and dried by lyophilization. Acetic acid (HAc) and sodium acetate (NaAc) were of HPLC grade. CD_3_COOD and D_2_O were of analytical grade. All other chemicals were of analytical grade. 

### 2.2. Fragmentation

#### 2.2.1. Kinetics

Purified original chitosan was dissolved in 0.05 M HCl with a constant stirring at room temperature for 12 h. Hydrochloric acid with concentration of 2.3 M was then added to chitosan solution. Final HCl and chitosan concentrations in the latter solution were 0.5 M and 1.0% (w/v), respectively. Hydrolytic fragmentation was carried out in a 1000 mL spherical flask equipped with a vertical condenser at 65°C for 30 h, while the solution was constantly stirred. A certain amount of the solution was drawn periodically and cooled down to 0°C immediately. One part of the solution was used to measure the solution viscosity at 25°C. Another part was used for recovery of chitosan fragments. The fragments have been used to measure intrinsic viscosity, the DA determination, and structural characterization. 

After fragmentation process, the fragments were recovered from chitosan suspension solutions as follows: the solution was neutralized with 1.0 N NaOH. The chitosan particles suspended in solution were precipitated by centrifugation, washed with deionized water, and recovered by lyophilization. 

#### 2.2.2. Effect of HCl Concentration

Reactions were performed as before except that final HCl concentration was varied between 0.1 and 1.0 M and reaction was terminated after 5 h. The reaction mixture was subsequently neutralized with 1.0 M NaOH to recover chitosan fragments. The recovery procedure was performed as described in Section 2.2.1.

### 2.3. Characterization

#### 2.3.1. Viscometry

Viscosities of chitosan solutions were measured using a capillary viscometer (Model AMV-200, Paar Physica USA Inc., Edison, NJ). Different capillaries having internal diameters of 0.9, 1.8, 3.0, and 4.0 mm were used to determine viscosity of chitosan solutions. The measurements were performed at inclination angle of 15° at 25°C. For solution viscosities higher than 800 cP, measurements were made with a rheometer using parallel plate geometry (RHOIS 902-30004, Rheometric Scientific Inc., Piscataway, NY, USA). The viscosities data was used to study kinetics of chitosan fragmentation.

#### 2.3.2. Intrinsic Viscosity Measurement

The intrinsic viscosities of the original chitosan and its fragments were measured in two solvents: (1) 0.1 M HAc/0.02 M NaCl (solvent A) and (2) 0.25 M HAc/0.25 M NaAc (solvent B) at 25°C using a capillary viscometer with an internal capillary diameter of 0.9 mm at inclination angle of 15°. These conditions along with the use of solution concentration lower than 1.0% (w/v) were selected, so corrections for kinetic energy and shear rate were negligible. Efflux times were measured for chitosan solutions (*t*_*s*_) and the solvent (*t*_0_). Measurement of efflux times was repeated four times and average efflux time was then converted to the ratio of *t*_*s*_/*t*_0_, which is proportional to relative viscosity of chitosan solution. The intrinsic viscosity was determined by both Huggins and Kraemer plots [29, 30]. The average value of two intercepts obtained from the two linear plots was considered as intrinsic viscosity of the polymer sample, [*η*]. The intrinsic viscosity data was used to calculate viscosity-average molecular weight of the polymer samples, *M*_*v*_. 

#### 2.3.3. Calculation of Viscosity-Average Molecular Weight and Number of Chain Scissions

The value of *M*_*v*_ was calculated in solvent A according to [31]

(1) $$\begin{array}{c} \left[\eta \right]=3.04\times 10^{-5}{M_{v}}^{1.26} \end{array}$$

and in solvent B was calculated using [32]

(2) $$\begin{array}{c} \left[\eta \right]=1.49\times 10^{-4}{M_{v}}^{0.79}. \end{array}$$

The average number of chain scissions, *α*, was calculated using the following equation [33]:

(3) $$\begin{array}{c} \alpha =\left(\frac{M_{v,o}}{M_{v,f}}\right)-1, \end{array}$$

where *M*_*v*,*o*_ and *M*_*v*,*f*_ are viscosity-average molecular weights of the original and the fragments, respectively.

#### 2.3.4. Size Exclusion Chromatography

Size exclusion chromatography (SEC) was used to compare molecular weight and molecular weight distribution of the original chitosan and its fragments. A HPLC/SEC instrument (Hewlett-Packard, Series 1050) was used with a refractive index detector, whose response is directly proportional to the polymer concentration in the eluting solution. Separation was achieved at 35°C using a TosoHaas-TSK gel column (GMPW_XL_, 30 cm × 7.8 mm) with 0.25 M HAc/0.25 M NaAc (solvent B for intrinsic viscosity measurement) as an eluent at a flow rate of 0.4 mL·min^−1^. 

#### 2.3.5. Structural Analysis

The chemical structure and the DA of the purified original chitosan and two fragments were determined by ^1^H NMR spectroscopy and elemental analysis. The procedures for structural analysis by the two techniques were the same as the procedures described by Hirai et al. [34] and Kasaai et al. [35].

## 3. Results and Discussion

### 3.1. Kinetics of Fragmentation

#### 3.1.1. General Considerations

Polymers containing heteroatoms in main and side chains are hydrolytically unstable [36]. Hydrolytic degradation of a polymer involves diffusion of the active medium into the polymer and reactions involving chemically unstable bonds. Two types of degradation by acid may take place in polymer molecules: (i) random and (ii) depolymerization. In the first type all hydrolytically unstable bonds are equally reactive, so that there should be no difference between a polymer and its simple analogue. In the second type the effective rate constant depends on the molecular weight. In practice the two types of degradation generally occur together [36]. The literature reports indicate that Both main chains (D-glycopyronsidic linkages) and side chains (N-acetyl) in chitin/ chitosan are susceptible to acid hydrolysis [37, 38].

Generally, the following two processes are taking place in acid hydrolysis of chitin/chitosan: the hydrolysis of the glycosidic linkages (main chain scission) and hydrolysis of N-acetyl linkages (side chain scission). The hydrolysis of the glycosidic linkages is SN_1_ reaction, whereas the hydrolysis of N-acetyl linkages is SN_2_ reaction [39]. At high temperature and in the presence of high concentration of acid both processes (scission of glycosidic linkages and scission of N-acetyl groups) occurred simultaneously [40]. The macromolecules decompose to the monomers, acetic acid, and other small molecules. This is because the degradation condition is aggressive and the energy required to break the two linkages is available at high temperature. 

#### 3.1.2. Evaluation of Experimental Results

**Figure 2:**
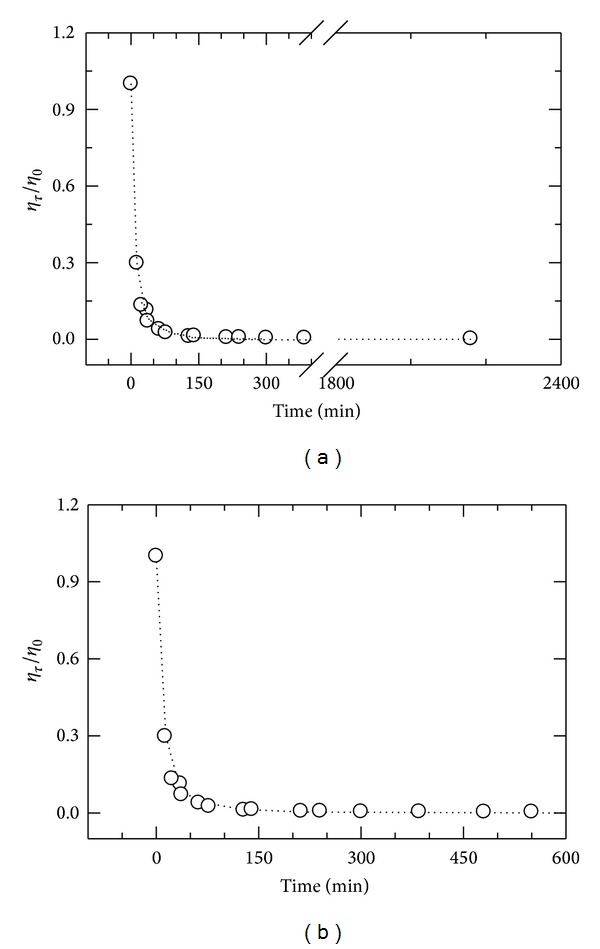
(a) The normalized solution viscosity, *η*_*τ*_/*η*_0_, as a function of reaction time (0–2400 min) for fragmentation of chitosan solution (1.0% w/v) by 0.5 N HCl. Open circle for experimental point and dotted line for curve fit; (b) the normalized solution viscosity, *η*_*τ*_/*η*_0_, as a function of reaction time (0–300 min) for fragmentation of chitosan solution (1.0% w/v) by 0.5 N HCl. Open circle for experimental point and dotted line for curve fit. The reaction was performed at 65°C, and solution viscosity was measured at 25°C.

Figure 2(a) shows the viscosity of reaction mixture normalized to initial viscosity (*η*_*t*_/*η*_0_) as a function of reaction time at 25°C. The solution viscosity dropped sharply at the beginning of the reaction (0–300 min), followed by a continuing slower decrease (300–2400 min). Since most of the fragmentation occurred within 5 h, the latter duration was selected as reaction time to study the effect of HCl concentration on chitosan fragmentation (chain scission, DA, and polydispersity). The reaction was best described by a double exponential decay function according to

(4) $$\begin{array}{c} \frac{\eta_{t}}{\eta_{0}}=A\cdot e^{-k_{1}\cdot t}+\left(1-A\right)\cdot e^{-k_{2}\cdot t}, \end{array}$$

where *k*_1_ and *k*_2_ are rate constants and *A* is amplitude of the first exponential function. The values of *A* (0.85), *k*_1_ (1.21 × 10^−1^), and *k*_2_ (2.03 × 10^−2^) suggest that about 85% of initial viscosity reduction was described by the first exponential decay. The solution viscosity as a function of reaction time within 0–300 min was illustrated in Figure 2(b). The values of constants (*A*, *k*_1_ and *k*_2_) for Figure 2(a) and for Figure 2(b) were nearly identical. 

**Figure 3:**
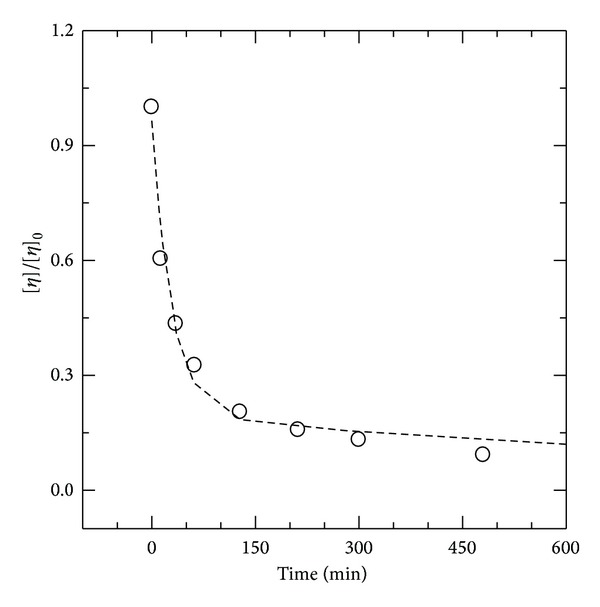
The normalized intrinsic viscosity of chitosan fragments, [*η*_*t*_]/[*η*_0_], as a function of reaction time (0–300 min) for fragmentation of chitosan solution (1.0% w/v) by 0.5 N HCl at 65°C. Open circle for experimental point and dotted line for curve fit. The reaction was performed at 65°C, and intrinsic viscosity was measured at 25°C.

Figure 3 shows the variation of intrinsic viscosity normalized to initial intrinsic viscosity ([*η*_*t*_]/[*η*_0_]) as a function of reaction time (0–300 min). The intrinsic viscosity as well as the viscosity-average molecular weight also dropped sharply at the beginning and within 0–300 min, followed by a continuing slower decrease (300–2400 min) (Table 1). The change in intrinsic viscosity can be also described by a double exponential decay function according to

(5) $$\begin{array}{c} \frac{\left[\eta_{t}\right]}{\left[\eta_{0}\right]}=B\cdot e^{-K_{1}\cdot \;t}+\left(1-B\right)\cdot {e^{-}}^{K_{2}\cdot \;t}\text{.} \end{array}$$

The first exponential decay described about 77% of reduction. The initial rate constant (*K*_1_ = 3.53 × 10^−2^) was greater than the second rate constant of the exponential decay (*K*_2_ = 8.1 × 10^−4^). Although solution viscosity (Figure 2(b)) and intrinsic viscosity (Figure 3) depicted similar trends, the values of *B* and rate constants (*K*_1_ and *K*_2_) for intrinsic viscosity were smaller than those of *A* and *k*_1_ and *k*_2_. This data indicates that (1) the reduction in solution viscosity was greater than that in intrinsic viscosity for the same time course; (2) larger macromolecules had a greater impact on solution viscosity than on intrinsic viscosity; and (3) all glycosidic linkages were not cleaved with the same rate. 

**Figure 4:**
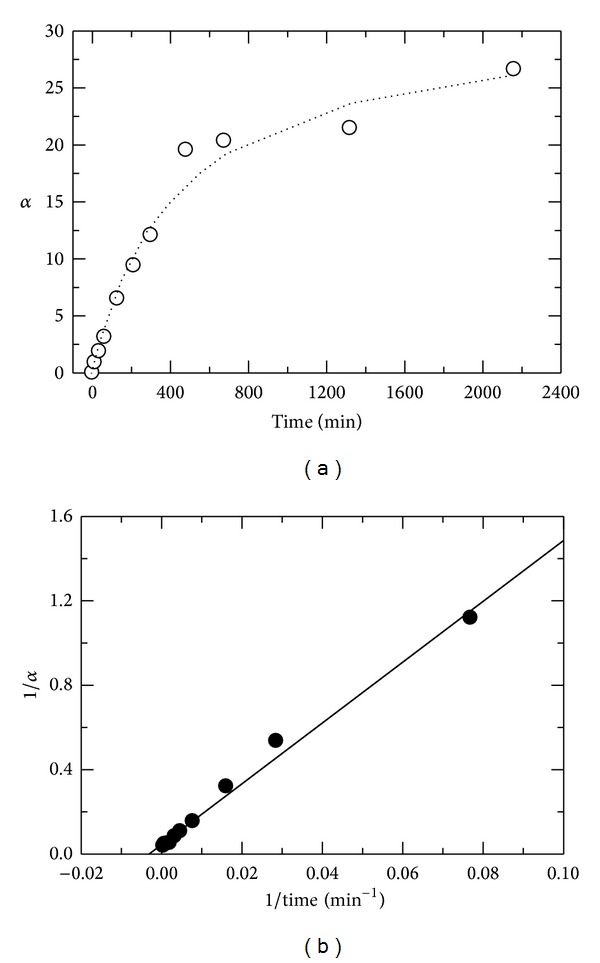
(a) Average number of chain scission, *α*, as a function of reaction time (0–2400 min) at 65°C. Open circle shows experimental points (O), and dotted line shows curve fit; (b) reciprocal of chain scission versus reciprocal of reaction time (0–2400 min). Chitosan concentration was 1.0% (w/v) in 0.1 M HAc.

Chain scission as a function of reaction time was hyperbolic (Figure 4(a)). The degradation was fast initially and gradually leveled off with an increase in irradiation time. The relationship between the reciprocal of chain scission versus the reciprocal of reaction time was linear (*R* ^2^ = 0.98) as follows:

(6) $$\begin{array}{c} \frac{1}{\alpha }=\frac{1}{\alpha_{\max}}+\frac{t_{R}}{\alpha_{\max}}\cdot \frac{1}{t}, \end{array}$$

where *α*_max⁡_ is maximum chain scission and *t*_*R*_ is reaction time at which the value of *α* equals to *α*_max⁡_/2.  Figure 4(b) illustrates 1/*α* versus 1/*t*. The values of *α*_max⁡_ and *t*_*R*_ were found to be 22.3 and 322 min, respectively. The value of *α*_max⁡_ = 22.3 corresponds to the limiting molecular weight of 87.5 kDa. The experimental results indicate that the reaction is of first order. 

The rate of fragmentation (−*dP*/*dt*) is proportional to the change in the number of moles for chitosan from the initial value (*m*/*M*_*o*_) to the final value (*m*/*M*_*f*_) at time, *t*:

(7) $$\begin{array}{c} -\frac{dP}{dt}≅-\frac{d\left(m/M\right)}{dt}=-\frac{md\left(1/M\right)}{dt}, \end{array}$$

where *M*_*o*_ and *M*_*f*_ are molecular weights of the original and the fragment obtained at reaction time, *t*, and *m* is the amount of chitosan in solution. At a constant concentration of chitosan, fragmentation increases with an increase in HCl concentration [*C*_*R*_] according to

(8) $$\begin{array}{c} -\frac{dP}{dt}=k{\left[C\right]}^{n}. \end{array}$$

**Figure 5:**
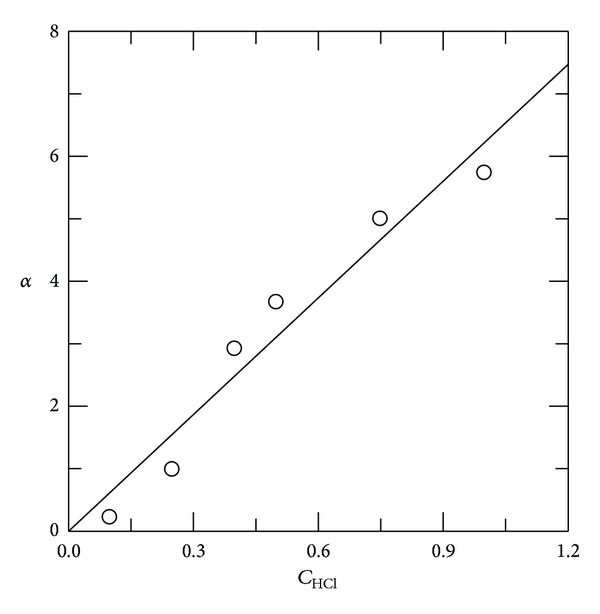
Average number of chain scissions, *α*, as a function of HCl concentration at 65°C. Chitosan concentration was 1.0% and reaction time was 5 h.

By combination of (7) and (8) and integration between *t* = 0 and *t* = *t*, one obtains

(9) $$\begin{array}{c} \frac{1}{M_{o}}\left\lfloor \frac{\left(M_{o}-M_{f}\right)}{M_{f}}\right\rfloor =k_{\text{app}}{\left[C_{R}\right]}^{n}\cdot t,\\ \alpha =k_{\text{app}}M_{o}{\left[C_{R}\right]}^{n}\cdot t, \end{array}$$

where *k*_app_ is the apparent rate constant and [(*M*_*o*_ − *M*_*f*_)/*M*_*f*_] or *α* is the average number of chain scissions and *n* is the order of reaction.  Figure 5 shows the effect of HCl concentration on *α* and the corresponding data are listed in Table 2. This data indicates that the fragmentation process progresses with an increase in HCl concentration. 

#### 3.1.3. Mechanism Proposed for Acid Hydrolysis of Chitin/Chitosan

In order to establish the mechanism of the degradation of polymeric materials in active media, one must know how the medium diffuses in the polymer. In polymers those dissolve readily in water, acids migrate with diffusion coefficients closely similar to that in aqueous solution [36]. Acid hydrolysis of the glycosidic linkages involves the following steps: (i) protonation of oxygen at glycosidic linkage; (ii) addition of water to the reducing sugar end group; and (iii) decomposition of protonated glycosidic linkages [22, 41–43]. The catalytic protons may be present in the water contained in the samples, and the protonated amino group of chitosan may probably also act as a proton donor in the catalysis [44]. Belamie et al. [45] studied hydrolysis of an original chitosan (DA% = 2.5) in solid state by means of either gaseous or concentrated aqueous HCl. They indicated that HCl acts as a reagent for the formation of chitosan hydrochloride, which is a necessary step to carry on the hydrolysis, and then as a catalyst in the hydrolysis reaction. The amine groups initially became protonated by H^+^ and then the excess value of acid catalyzes the reaction. 

**Scheme 1:**
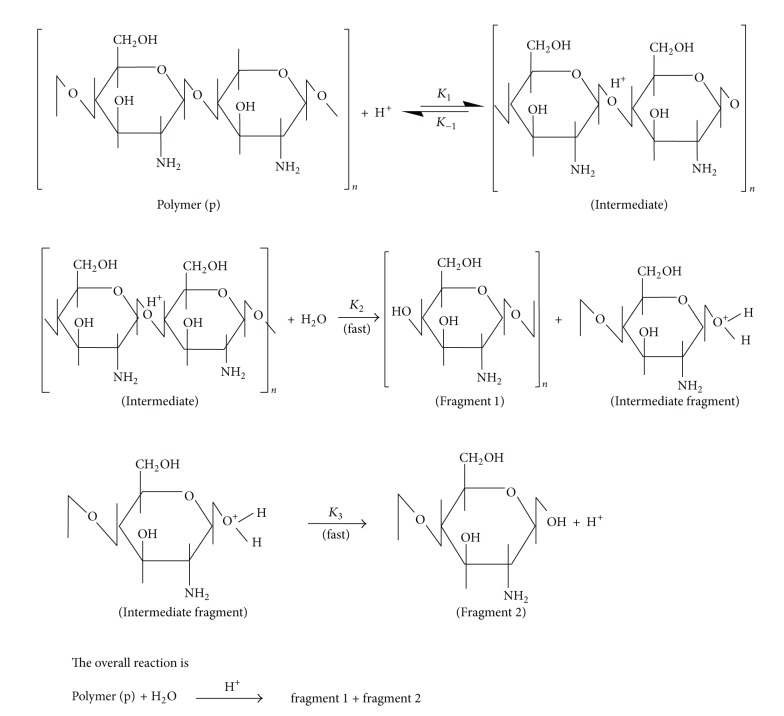
Mechanism hydrolysis of chitosan by an acid.

Under the experimental conditions, the following mechanism is adapted with the experimental data. Fragmentation involves two steps: (1) protonation of glycosidic linkages and (2) splitting of large macromolecular chains into two smaller ones. Therefore, fragmentation is initiated by attachment of a proton (H_3_O^+^) to the glycoside linkage, followed by scission of larger macromolecules into smaller ones (see Scheme 1).

The rate of fragmentation based on the above mechanism can be described by the following equations. For convenience, the concentrations of intermediate, intermediate fragment, and chitosan denoted by [*I*1], [*I*2], and [*P*], respectively:

(10) $$\begin{array}{c} -\frac{dP}{dt}=K_{1}\left[{\text{H}}^{+}\right]\left[P\right]-K_{-1}\left[I1\right], \end{array}$$

(11) $$\begin{array}{c} -\frac{d\left[I1\right]}{dt}=-K_{-1}\left[{\text{H}}^{+}\right]\left[P\right]+K_{-1}\left[I1\right]+K_{2}\left[I1\right]\left[{\text{H}}_{2}\text{O}\right], \end{array}$$

(12) $$\begin{array}{c} -\frac{d\left[I2\right]}{dt}=-K_{2}\left[I1\right]\left[{\text{H}}_{2}\text{O}\right]+K_{3}\left[I2\right]. \end{array}$$

Assume steady-state conditions for (11) and (12) yield in

(13) $$\begin{array}{c} \left[I1\right]=\frac{K_{1}\left[{\text{H}}^{+}\right]\left[P\right]}{\left(K_{-1}+K_{2}\left[{\text{H}}_{2}\text{O}\right]\right)},\\ \left[I2\right]=\frac{K_{2}\left[I1\right]\left[{\text{H}}_{2}\text{O}\right]}{K_{3}}. \end{array}$$

Substitution of [*I*1] and [*I*2] into (10) results in (14) or (15) as follows:

(14) $$\begin{array}{c} -\frac{dP}{dt}=K_{1}\left[P\right]\left[{\text{H}}^{+}\right]-K_{-1}\times \left[\frac{K_{1}\left[{\text{H}}^{+}\right]\left[P\right]}{\left(K_{-1}+K_{2}\left[{\text{H}}_{2}\text{O}\right]\right)}\right], \end{array}$$

(15) $$\begin{array}{c} -\frac{dP}{d}=K_{1}\left[P\right]\left[{\text{H}}^{+}\right]\left[1-\frac{K_{-1}}{\left(K_{-1}+K_{2}\left[{\text{H}}_{2}\text{O}\right]\right)}\right]. \end{array}$$

All parameters in the right hand side of (15) are constants except the value of [H^+^]. The constants can be replaced by a constant *K*, yielding

(16) $$\begin{array}{c} -\frac{dP}{dt}=K\left[{\text{H}}^{+}\right]. \end{array}$$

The value of *dP* is proportional to the number of chain scissions, *α*; therefore, we can write

(17) $$\begin{array}{c} -dP\propto \alpha =K\cdot \left[{\text{H}}^{+}\right]\cdot t. \end{array}$$

The above mechanism is in accordance with the kinetics data (Figures 4 and 5 and (17)) and in agreement with several reported data [22, 27, 40, 46–48].

#### 3.1.4. The Rate of Reaction for Acid Hydrolysis of Chitin/Chitosan

The rate of fragmentation depends on molecular weight of the original chitin/chitosan (see Figures 2 and 3). Chain scission, reduction in solution viscosity, and reduction in intrinsic viscosity decreased with an increase in reaction time (see Figures 2, 3, and 4). The *M*_*v*_ of chitosan decreased from 2038 kDa to 73.8 kDa after 30 h of acid hydrolysis (see Table 2). Värum et al. [40] reported that the rate of acid hydrolysis of the glycosidic linkages was similar to the rate of deacetylation at low concentration of HCl, and was more than 10 times higher than the rate of deacetylation in concentrated acid. The order of acid hydrolysis was A-A *≈* A-D ≫ D-A *≈* D-D [27, 40]. A wide range of molecular weights of chitosans (from *M*_*v*_ = 71 kDa to <214 kDa) were obtained in homogeneous hydrolysis of chitosan using 85% H_3_PO_4_ at different reaction times (1–840 h) and temperatures (40–80°C) using initial chitosan (*M*_*v*_ = 214 kDa) [28]. The rate of acid hydrolysis of chitosan in 85% H_3_PO_4_ was maximum initially and then decreased slowly [28].

Hydrolysis of dimer of chitin [GlcNAc (1–4) GlcNAc] [24] and tetramers of fully N-acetylated chitin (GlcNAc) and fully N-deacetylated chitosan (GlcN) [25] by different HCl concentrations of HCl (3–12 M) and temperatures (20–35°C) have been studied. They reported that the hydrolysis of the glycosidic linkages was SN_1_ reaction and the rate limiting is the formation of the carbonium ion. The hydrolysis of the N-acetyl linkage is SN_2_ reaction. The rate-limiting step is addition of water to the carbonium ion. The initial rate constant of thermal degradation of chitosan chlorides in the solid state increased with an increase in H^+^ concentration [44]. The rate-determining step was the formation of the activated complex (a cyclic carbonium-oxonium ion) after the protonation step [44]. Belamie et al. [45] indicated that the kinetics of chitosan degradation in solid state by HCl depend on both the degree of hydration and HCl concentration. The more the HCl, the faster the reaction. Rupley [49] noted that the rate of hydrolysis depends on the structure of chitin/chitosan and energy of activation of the two hydrolysis processes (glycosidic linkages and N-acetyl linkages). 

### 3.2. The Effects of Reaction Conditions on Molecular Weight, Polydispersity, and DA of Chitosan Fragments

We did not observe any change in intrinsic viscosity as well as molecular weight of chitosan when the reaction was performed in acetic acid under given experimental conditions (pH > 3.5; time course = 300 min; *T* = 65°C). On the other hand, acid hydrolysis takes place when pH of solution is lower than 3.5. Belamie et al. [45] also reported that no hydrolysis takes place when pH is above 4.5. 

**Figure 6:**
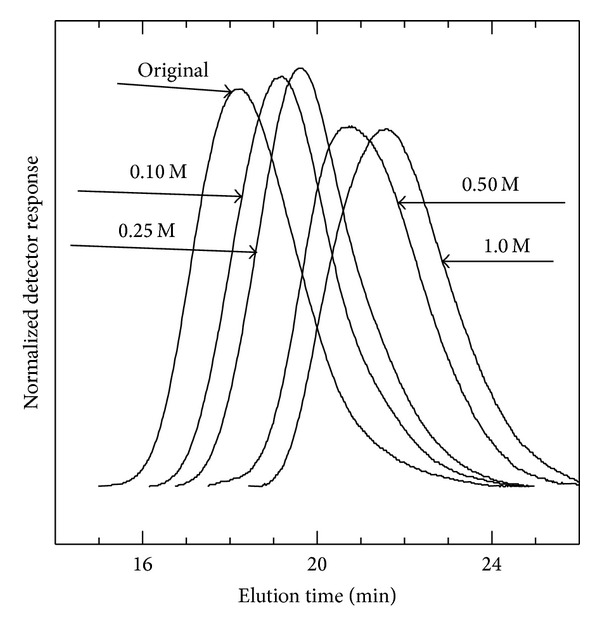
Size exclusion chromatograms of the original chitosan and the fragments resulting from hydrolysis of 1.0% (w/v) chitosan with different HCl concentrations at 65°C for 5 h.

Molecular weight and polydispersity of the original and resulting fragments at different HCl concentrations were analyzed.  Figure 6 shows SEC chromatograms of the original chitosan and its fragments. The chromatograms were shifted from a low elution volume (corresponding to the original chitosan) towards higher elution volumes with an increase in HCl concentration. These authors reported that the polydispersity of the fragments did not change significantly compared to that of the original ones. Smidsrød et al. [26] also reported that the polydispersity of chitosan in acid hydrolysis did not vary as a function of reaction time. On the contrary, other investigators [48, 50–53] obtained chitosan fragments with a wider polydispersity compared to the original chitosans. These authors reported that the polydispersity of the fragments increased with an increase in HCl concentration, temperature, and reaction time. The polydispersity of the fragments depends on the sequence of commoner units of the original polymer. Our experimental conditions (0.0 < *C*_HCl_ ≤ 1.0, temperature = 65°C) were roughly soft compared to the most reported literature experimental conditions. The values of *α* and polydispersity of the resulting fragments depend also on the initial molecular weight of chitosan. 

**Figure 7:**
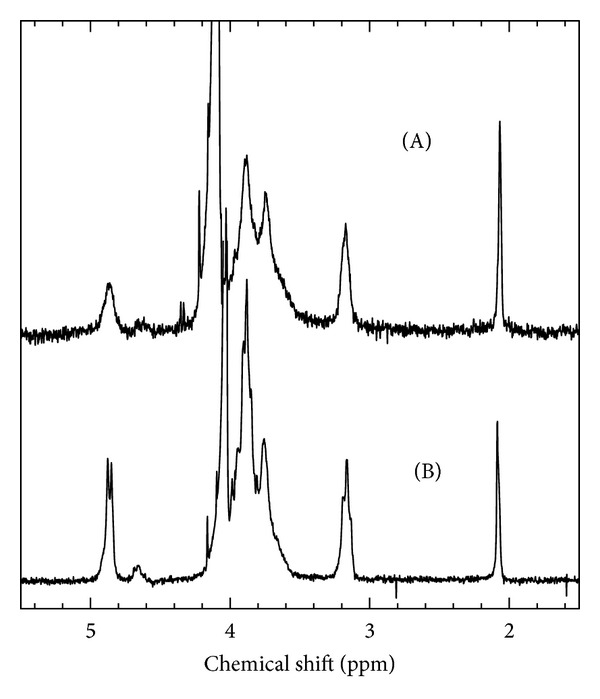
^1^H NMR spectra of (A) original chitosan and (B) the fragment in 2% (w/w) CD_3_COOD/D_2_O at 70°C. The fragment obtained from hydrolysis (sample F19, Table 2).

The chemical structures of the original chitosan and a fragment were analyzed by ^1^H NMR spectroscopy.  Figure 7 shows the NMR spectra of the original chitosan (spectrum A) and the fragment obtained from HCl hydrolysis (spectrum B). NMR spectrum did not show any essential difference in comparison with the original polymer, except for the lower resolution of the original chitosan, which is commonly observed for high molecular weight samples. The values of DA determined by ^1^H NMR and elemental analysis are presented in Table 3. The DA decreased slightly with fragmentation process, suggesting that a limited hydrolysis of N-acetyl groups has occurred. Li et al. [54] reported that the DA of the samples obtained from acid hydrolysis was decreased compared to the original chitosan. Värum et al. [40] reported that the rate of hydrolysis of the glycosidic linkages equals to the rate of N-deacetylation in dilute HCl, whereas in concentrated HCl the rate of glycosidic linkages is more than 10 times faster than that of N-acetyl linkages. The experimental conditions of the present study were appropriated to achieve the DA of fragments slightly smaller than that of the original ones. 

### 3.3. Estimation of Molecular Weight of Fragments Using Proposed Equations

**Figure 8:**
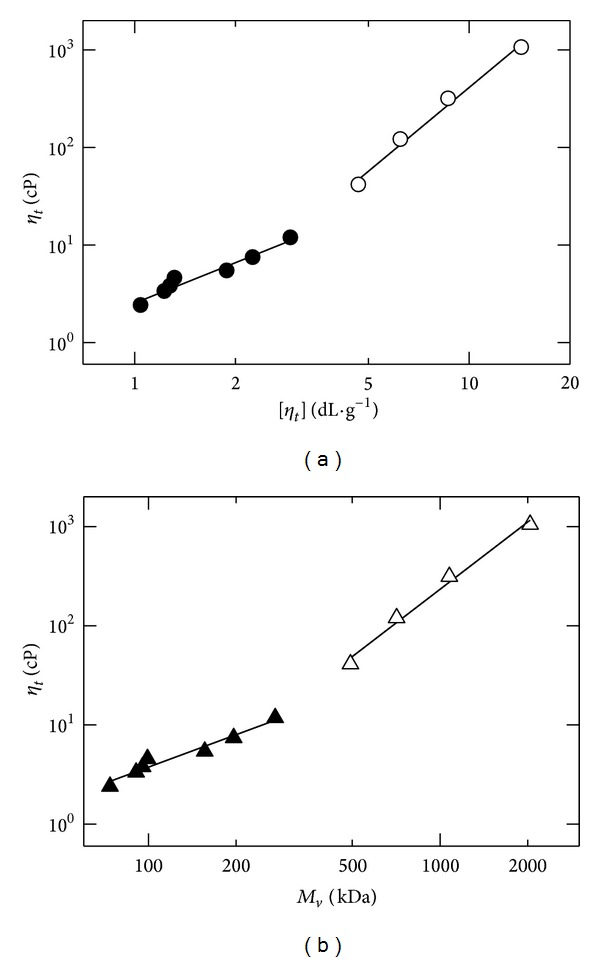
(a) Viscosity of reaction mixture at time *t*, *η*_*t*_, versus intrinsic viscosity of resulting fragment, [*η*_*t*_]; (b) viscosity of reaction mixture at time *t*, *η*_*t*_, versus viscosity-average molecular weight of resulting fragment, *M*_*v*_, for fragmentation of 1.0% (w/v) chitosan solution by 0.5 N HCl at 65°C.

Figure 8(a) shows relative solution viscosity (*η*_*r*_) of the reaction mixture versus intrinsic viscosity of the fragments. The relationship was bilinear. The slope of the initial linear portion was smaller (initial slope = 1.38 and final slope = 2.85), and the change in slope occurred after *c*[*η*] > 4.5 (see (18) and (19)). The plot of *η*_*r*_ as a function of viscosity-average molecular weight was also bilinear, with smaller slopes (initial slope = 1.09 and final slope = 2.26), and change in the slope occurred above *M*_*v*_ > 450 kDa ((20) and (21)):

(18) $$\begin{array}{c} \log\eta_{r}=0.4+1.38\log\left[\eta \right]\;c\left[\eta \right]<4.5, \end{array}$$

(19) $$\begin{array}{c} \log\eta_{r}=-0.24+2.85\log\left[\eta \right]\;c\left[\eta \right]>4.5, \end{array}$$

(20) $$\begin{array}{c} \log\eta_{r}=-1.6+1.09\log M_{v}\;M_{v}<450\;\text{kDa}, \end{array}$$

(21) $$\begin{array}{c} \log\eta_{r}=-4.4+2.25\log M_{v}\;M_{v}>450\;\text{kDa}. \end{array}$$

Large macromolecules contribute more impact to solution viscosity and intrinsic viscosity compared to small macromolecules. Entanglements begin to occur when the product of intrinsic viscosity and polymer concentration exceed unity (*c*[*η*] > 1) [55, 56]. The level of overlapping between macromolecular chains increases with an increase in solution concentration and intrinsic viscosity or the product of solution concentration and intrinsic viscosity (*c*[*η*]). In dilute and semidilute regions, the slopes of plots are smaller than those of concentrated region. The higher values of slopes for upper portions of Figures 8(a) and 8(b) indicate a greater level of polymer-polymer interactions and lower level of the polymer-solvent interactions. The ratio (upper slope/lower slope) was found to be 2.1 for both Figures 8(a) and 8(b). The linear plots were obtained for each portion (lower or upper) of the two figures. They can be used to estimate intrinsic viscosity or viscosity-average molecule weight of chitosan fragments from measurements of their solution viscosity of corresponding reaction mixture at a given time (*t*). Equations (18)–(21) can be applied to chitosans having similar DA value and most probably similar distribution of the DA values between the individual chains of the chitosan. The advantages of employing these equations over intrinsic viscosity measurements are that determination of solution viscosity needs only a single measurement, whereas the determination of intrinsic viscosity needs several solution viscosities measurements, which is timeconsuming. 

## 4. Conclusions

A double exponential decay function best described the fragmentation of chitosan by HCl; that is, reduction in solution viscosity or reduction in intrinsic viscosity versus reaction time was followed by an exponential decay function. The rate of the fragmentation was fast initially, and further fragmentation was a slow process. A mechanism for the fragmentation process based on kinetics data was proposed. Chitosan fragmentation proceeds by consecutive reactions. The number of chain scission increased with an increase in concentration of HCl. The polydispersity of the fragments did not increase significantly compared to the original chitosan. ^1^H NMR spectral analysis and elemental analysis demonstrated that the DA of the fragments slightly decreased with an increase in reaction time or acid concentration. 

An easy method to estimate intrinsic viscosity as well as viscosity-average molecular weight (through MHS equation) from a single measurement of solution viscosity of the reaction mixture was also proposed. The molecular weight of the fragments decreased as a function of time and acid concentration. 

## Figures and Tables

**Table 1:** Intrinsic viscosity, (*η*), of chitosans in 0.25 M HAc/0.25 M NaAc, viscosity-average molecular weight, *M*_*v*_, and average number of chain scissions, *α*, for the fragments prepared by hydrolysis fragmentation of 1.0% chitosan at different reaction times with 0. 5 M HCl at 65°C.

Chitosan sample	Reaction time (min)	(*η*) (dL/g)	*M*_*v*_ (kDa)	*α*
Original	—	14.37	2038	—
F1	13	8.68	1076	0.89
F2	35	6.25	710	1.87
F3	62	4.68	493	3.13
F4	128	2.93	272	6.49
F5	212	2.26	196	9.42
F6	300	1.89	156	12.06
F7	480	1.32	99.2	19.50
F8	675	1.28	95.5	20.45
F9	1320	1.23	90.8	21.44
F10	2160	1.05	73.8	26.24

**Table 2:** The intrinsic viscosity, (*η*), the viscosity-average molecular weight, *M*_*v*_, and the average number of chain scissions (*α*) for the fragments prepared by hydrolysis of 1.0% chitosan with different HCl concentrations at 65°C for 5 h.

Chitosan sample	(HCl) (M)	(*η*) (dL/g)	*M*_*v*_ (kDa)	*α*
Original	—	25.86	1965	—
F11	0.10	20.20	1616	0.217
F12	0.25	10.92	991.5	0.982
F13	0.40	4.33	475.9	3.13
F14	0.40	4.98	531.7	2.70
F15	0.50	3.92	439.7	3.47
F16	0.50	3.54	405.5	3.85
F17	0.75	2.71	328.1	4.99
F18	1.00	2.29	287.0	5.85
F19	1.00	2.39	296.9	5.62

**Table 3:** Degree of acetylation (DA) for original chitosan and two fragments as determined by elemental analysis (E. A.) or ^1^H NMR.

Chitosan sample	DA (%)	
	E. A.	NMR
Original	26	26
(F12, Table 2)	21	—
(F19, Table 2)	19	18

## References

[1] Chatelet C, Damour O, Domard A (2001). Influence of the degree of acetylation on some biological properties of chitosan films. *Biomaterials*.

[2] Shahidi F, Arachchi JKV, Jeon Y (1999). Food applications of chitin and chitosans. *Trends in Food Science and Technology*.

[3] Sandford PA, Steinnes A, Shalaby SW, McCormick CL, Butler GB (1991). Biomedical applications of high-purity chitosan, physical, chemical and bioactive properties. *Water-Soluble Polymers, Synthesis, Solution Properties and Applications*.

[4] Sandford PA, Hutchings GP, Yalpani M (1987). Chitosan—a natural, cationic biopolymer: commercial applications. *Industrial Polysaccharides, Genetic Engineering, Structure/Property Relations and Applications*.

[5] Knorr D (1984). Use of chitinous polymers in food. *Food Technology*.

[6] Jeon Y, Kamil JYVA, Shahidi F (2002). Chitosan as an edible invisible film for quality preservation of herring and Atlantic cod. *Journal of Agricultural and Food Chemistry*.

[7] Makino Y, Hirata T (1997). Modified atmosphere packaging of fresh produce with a biodegradable laminate of chitosan-cellulose and polycaprolactone. *Postharvest Biology and Technology*.

[8] Cruz Z, Lauzon HL, Olabarrieta JC Impact of chitosan on growth inhibition of micro-organisms isolated from fishery products.

[9] Knorr D (1991). Recovery and utilization of chitin and chitosan in food processing waste management. *Food Technology*.

[10] El Ghaouth A, Arul J, Ponnampalam R, Boulet M (1991). Use of chitosan coating to reduce weight loss and maintain quality of cucumbers and bell peppers. *Journal of Food Processing and Preservation*.

[11] El Ghaouth A, Arul J, Ponnampalam R, Boulet M (1991). Effect of chitosan coating on the storability and quality of fresh strawberries. *Journal of Food Science*.

[12] El Ghaouth A, Ponnampalam R, Castaigne F, Arul J (1991). Chitosan coating to extend the storage life of mature green tomatoes. *HortScience*.

[13] Reddy MVB, Belkacemi K, Corcuff R, Castaigne F, Arul J (2000). Effect of pre-harvest chitosan sprays on post-harvest infection by *Botrytis cinerea* quality of strawberry fruit. *Postharvest Biology and Technology*.

[14] Rathke TD, Hudson SM (1993). Determination of the degree of N-deacetylation in chitin and chitosan as well as their monomer sugar ratio by near infrared spectroscopy. *Journal of Polymer Science A*.

[15] Synowiecki J, Al-Khateeb NA (2003). Production, properties, and some new applications of chitin and its derivatives. *Critical Reviews in Food Science and Nutrition*.

[16] Wu ACM, Bough WA, Muzzarelli RAA, Pariser ER (1978). A study of variables in the chitosan manufactering process in relation to molecular weight distribution, chemical characteristics and waste treatment. *Proceedings of the 1st International Conference on Chitin/Chitosan*.

[17] Allan GG, Altman LC, Bensinger RE, Zikakis JP (1984). Biomedical applications of chitin and chitosan. *Chitin, Chitosan and Related Enzymes*.

[18] Hadwiger LA, Fristensky B, Riggleman RC, Zikakis JP (1984). Chitosan, a natural regulator in plant-fungal pathogen interactions, increases crop yields. *Chitin, Chitosan, and RelatEd Enzymes*.

[19] Olsen R, Schwartzmiller D, Weppner W, Winandy R, Skjak-Braek G, Anthonsen T, Sandford P (1989). Biomedical applications of chitin and its derivatives. *Chitin and Chitosan*.

[20] Tokura S, Miura Y, Johmen M, Nishi N, Nishimura S-I (1994). Induction of drug specific antibody and the controlled release of drug by 6-O-carboxymethyl-chitin. *Journal of Controlled Release*.

[21] Kasaai MR, Charlet G, Arul J (2000). Master curve for concentration dependence of semi-dilute solution viscosity of chitosan homologues: the Martin equation. *Food Research International*.

[22] Kikkwa Y, Kawada T, Furukawa I, Sakuno T (1990). A convenient preparation method of chito-oligosaccharides by acid hydrolysis. *Journal of Faculty of Agriculture, Tottori University*.

[23] Scheel O, Thiem J, Muzzarelli RAA, Peter MG (1997). Cleavage of chitin by means of aqueous hydrochloric acid and isolation of chito-oligosaccharides. *Chin Handbook*.

[24] Einbou A, Vårum KM (2007). Depolymerization and de-N-acetylation of chitin oligomers in hydrochoric acid. *Bio-Macromolecules*.

[25] Einbu A, Grasdalen H, Vårum KM (2007). Kinetics of hydrolysis of chitin/chitosan oligomers in concentrated hydrochloric acid. *Carbohydrate Research*.

[26] Smidsrød O, Ottøy MH, Anthonsen MW, Värum KM, Domard A, Roberts GAF, Värum KM (1997). Solution properties of chitosan. *Advances in Chitin Science, Volume II*.

[27] Vårum KM, Ottøy MH, Smidsrød O (2001). Acid hydrolysis of chitosans. *Carbohydrate Polymers*.

[28] Jia Z, Shen D (2002). Effect of reaction temperature and reaction time on the preparation of low-molecular-weight chitosan using phosphoric acid. *Carbohydrate Polymers*.

[29] Flory PJ (1953). *Principles of Polymer Chemistry*.

[30] Tanford C (1961). *Physical Chemistry of Macromolecules*.

[31] Roberts GAF, Domszy JG (1982). Determination of the viscometric constants for chitosan. *International Journal of Biological Macromolecules*.

[32] Kasaai MR, Arul J, Charlet G (2000). Intrinsic viscosity-molecular weight relationship for chitosan. *Journal of Polymer Science B*.

[33] Nemzek TL, Guillet JE (1977). Calculation of accuracy and correction factors in the viscometric determination of chain scission in polymers. *Macromolecules*.

[34] Hirai A, Odani H, Nakajima A (1991). Determination of degree of deacetylation of chitosan by ^1^H NMR spectroscopy. *Polymer Bulletin*.

[35] Kasaai MR, Arul J, Chin SL, Charlet G (1999). The use of intense femtosecond laser pulses for the fragmentation of chitosan. *Journal of Photochemistry and Photobiology A*.

[36] Zaikov GE (1975). Kinetic study of the degradation and stabilization of polymers. *Russian Chemical Reviews*.

[37] Timell TE (1964). The acid hydrolysis of glycosides. I. General conditions and the effect of the nature of the aglycone. *Canadian Journal of Chemistry*.

[38] Ondera K, Komano T (1961). Acid hydrolysis of Methyl 2-amino-2-deoxy-D-glucopyranosides. *Agricultural Biological Chemistry*.

[39] Niola F, Basora N, Chornet E, Vidal PF (1993). A rapid method for the determination of the degree of N-acetylation of chitin-chitosan samples by acid hydrolysis and HPLC. *Carbohydrate Research*.

[40] Värum KM, Koga D, Smidsrød O, Uragami T, Kurita K, Fukamizo T (2001). Degradation of chitosans. *Chitin and Chitosan, Chitin and Chitosan in Life Science*.

[41] BeMiller JM, Wolfram ML (1967). Acid-catalyzed hydrolysis of glycosides. *Advances in Carbohydrate Chemistry*.

[42] Lazár M, Bleha T, Rychlý J (1989). *Chemical Reactions of Natural and Synthetic Polymers*.

[43] Feather MS, Harris JF (1965). The acid-catalyzed hydrolysis of glycopyranosides. *Journal of Organic Chemistry*.

[44] Holme HK, Foros H, Pettersen H, Dornish M, Smidsrød O (2001). Thermal depolymerization of chitosan chloride. *Carbohydrate Polymers*.

[45] Belamie E, Domard A, Giraud-Guille M (1997). Study of the solid-state hydrolysis of chitosan in presence of HCl. *Journal of Polymer Science A*.

[46] Värum KM, Ottøy MH, Smidsrød O (1994). Water-solubility of partially N-acetylated chitosan as a function of pH: effect of chemical composition and depolymerization. *Carbohydrate Polymers*.

[47] Duoxim S, Yan Z, Anjie D, Goosen MFA, Sun AM, Feng H, Han Y, Huang L (1991). Studies on the degradation of chitosan and preparation of chitosan-alginate microcapsules. *Polymers and Biomaterials, International Symposium Proceedings*.

[48] Rogozhin SV, Gamzazade AI, Chlenov MA, Leonova YY, Sklyar AM, Dotdayev SK (1988). The partial acidic hydrolysis of chitosan. *Polymer Science USSR*.

[49] Rupley JA (1964). The hydrolysis of chitin by concentrated hydrochloric acid, and the preparation of low-molecular-weight substrate for lysozyme. *Biochime et Biophysique Acta*.

[50] Nordttveit RJ, Vårum KM, Smidsrød O (1994). Degradation of fully water-soluble, partially N-acetylated chitosans with lysozyme. *Carbohydrate Polymers*.

[51] Boryniec S, Strobin G, Struszczyk H, Niekraszewicz A, Kucharska M (1997). GPC studies of chitosan degradation. *International Journal of Polymer Analysis and Characterization*.

[52] Hirano S, Tsuchida H, Nagao N (1989). N-acetylation in chitosan and the rate of its enzymic hydrolysis. *Biomaterials*.

[53] Berger LR, Weiser RS (1957). The *β*-glucosaminidase activity of egg-white lysozyme. *Biochimica et Biophysica Acta*.

[54] Li J, Revol J-F, Marchessault RH (1997). Effect of degree of deacetylation of chitin on the properties of chitin crystallites. *Journal of Applied Polymer Science*.

[55] Graessley WW (1974). The entanglement concept in polymer rheology. *Advances Polymer Science*.

[56] Hager BL, Berry GC (1982). Moderately concentrated solutions of polystyrene. 1. Viscosity as a function of concentration, temperature, and molecular weights. *Journal of Polymer Science B*.

